# Intestinal and epidermal-specific analysis of ZIP-1 function and expression in *Caenorhabditis elegans*

**DOI:** 10.17912/micropub.biology.002114

**Published:** 2026-04-16

**Authors:** James A. Tirtorahardjo, Vladimir Lažetić, Emily R. Troemel

**Affiliations:** 1 Department of Cell and Developmental Biology, UC San Diego, San Diego, CA, US; 2 Department of Biological Sciences, The George Washington University, Washington, DC, US

## Abstract

In the nematode *Caenorhabditis elegans* , infection with intracellular intestinal pathogens, including microsporidia and the Orsay virus, activates a transcriptional immune response called the intracellular pathogen response (IPR). Upregulation of about 1/3 of IPR genes requires a bZIP transcription factor ZIP-1. Previous work has demonstrated that ZIP-1 promotes anti-viral immunity, but how ZIP-1 protein is regulated and where it controls immune defense remains unclear. Here we show that intestinal-specific rescue of ZIP-1 drives IPR gene expression and promotes resistance to viral infection. We also show that proteasome blockade increases ZIP-1::GFP protein levels independently of the *zip-1* promoter.

**Figure 1. Analysis of tissue-specific ZIP-1::GFP expression and function:** **A)** Diagram of *zip-1 ::gfp* constructs. Top: *vha-6p* drives intestinal expression, Middle: *dpy-7p* drives epidermal expression, Bottom: endogenous tag. 100 bp scale bars. **B)** Fluorescent micrographs of ZIP-1 ::GFP expression in a wild-type background after 4 hours of bortezomib treatment. Merge of GFP in green, and autofluorescence in blue. **C)** Fluorescent micrographs of ZIP-1 ::GFP expression in a *zip-1 (jy13)* background after 4 hours of bortezomib treatment. Merge of GFP in green, autofluorescence in blue, and bright-field Nomarski. **D)** Time course of ZIP-1 ::GFP nuclear expression during bortezomib treatment. Average of 3 independent experiments shown, with 20 worms per experiment. Error bars are standard deviation (SD). **E)** IPR gene activation as quantified by qRT-PCR. Animals treated at the L4 stage with bortezomib for 30 minutes, normalized against a DMSO-treated wild-type control. Average of 3 to 6 independent experiments shown, with each experiment including 10,000 animals per replicate. Welch's T-test, unpaired, one-tailed * p <0.05. Error bars are standard error of the mean (SEM). **F)** Orsay viral load as quantified by qRT-PCR of Orsay RNA1. Animals infected at the L4 stage (see Methods), quantified at 24 hpi. Average of 8 independent experiment, with each experiment including 2000 infected animals per sample. Welch's T-test, unpaired, one-tailed * p <0.05 , ****p <0.0001. Error bars are SD. **B-E)** All bortezomib treatments were at final concentration of 20 µM bortezomib compared to DMSO vehicle control. **B, C)** Arrows indicate intestinal nuclei, arrowheads indicate epidermal nuclei. 25 µm scale bars.

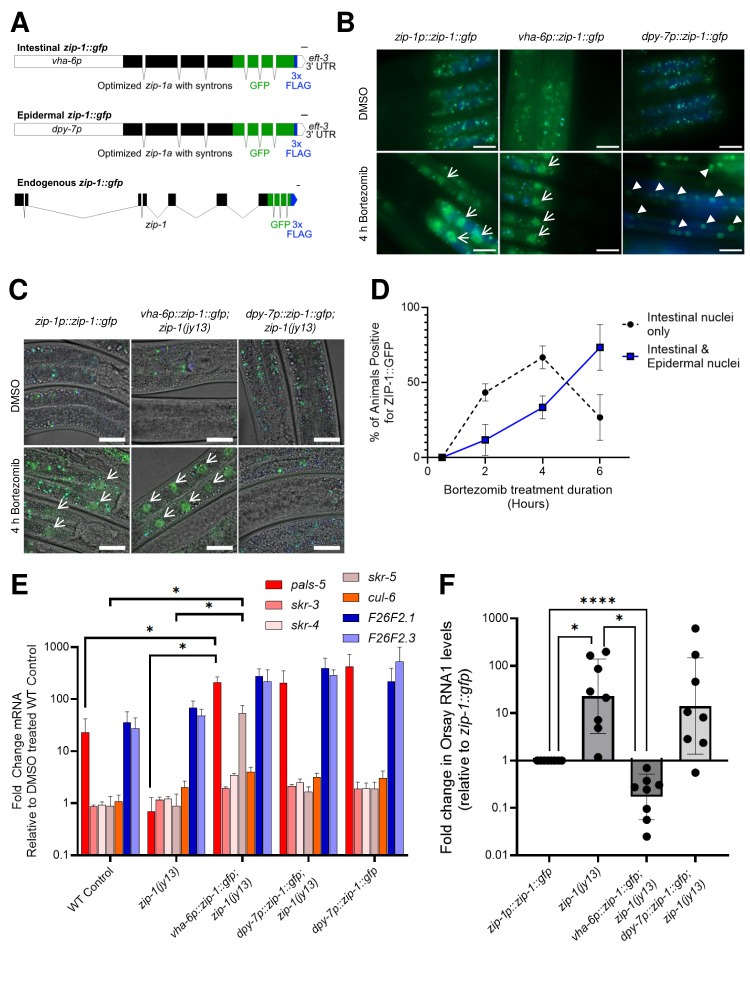



## Description

Transcriptional induction of immune genes needs to be carefully controlled to promote defense against pathogen infection, without causing collateral damage to the host. In the nematode *C. elegans* , infection with natural pathogens of the intestine, including species of microsporidia (intracellular fungi) and a single-stranded RNA virus known as the Orsay virus , activates a transcriptional innate immune program called the intracellular pathogen response (IPR) &nbsp;(Bakowski et al., 2014; Castiglioni et al., 2024; Chen et al., 2017; Reddy et al., 2019; Sarkies et al., 2013). The IPR promotes defense against infection, but IPR overactivation can slow organismal development and shorten lifespan (Reddy et al., 2019). ZIP-1 is a bZIP transcription factor that controls induction of about 1/3 of IPR genes, and promotes defense against viral infection (Lazetic et., 2022). How ZIP-1 protein expression is regulated and where ZIP-1 acts to control immunity have not been carefully explored.

&nbsp;

Our previous data indicate that ZIP-1 can be expressed in the intestine or the epidermis, depending on the trigger. Specifically, an endogenously tagged ZIP-1 ::GFP protein (hereafter referred to as *zip-1 p::* ZIP-1 ::GFP) is not visible under baseline conditions, but upon infection with microsporidia or the Orsay virus becomes visible in intestinal nuclei (Lazetic et al., 2022). *zip-1 p::* ZIP-1 ::GFP is also visible in intestinal nuclei after 4 hours (h) of treatment with the proteasome inhibitor bortezomib, which induces mRNA expression of IPR genes, as well as *zip-1* mRNA expression. In contrast, *zip-1 p::* ZIP-1 ::GFP is expressed prominently in epidermal nuclei when the IPR is genetically activated by loss of the IPR inhibitor PALS-22 (Gang et al., 2022). To better understand the roles ZIP-1 has in the intestine and the epidermis, we developed tissue-specific ZIP-1 expression strains. We generated single-copy transgenic strains with ZIP-1 ::GFP expression under the control of the *vha-6p* intestinal-specific promoter or the *dpy-7p* epidermal-specific promoter. ZIP-1 expression in these strains was compared against *zip-1 p::* ZIP-1 ::GFP ( Figure 1A ). Given that *zip-1* mRNA is also upregulated by IPR triggers, these tissue-specific ZIP-1 strains also allowed us to investigate whether ZIP-1 protein levels might be induced by IPR triggers independently of the *zip-1* promoter.

**&nbsp;**

Similar to *zip-1 p* :: ZIP-1 ::GFP, we observed no expression of *vha-6p* :: ZIP-1 ::GFP or *dpy-7p* :: ZIP-1 ::GFP under baseline conditions ( Figure 1B ). However, when we treated animals with bortezomib for 4 h, we saw *vha-6p* :: ZIP-1 ::GFP expression in intestinal nuclei, and *dpy-7p* :: ZIP-1 ::GFP expression in epidermal nuclei after bortezomib treatment ( Figure 1B ). The bortezomib-induced expression of nuclear ZIP-1 ::GFP in the tissue-specific strains is consistent with our observations using endogenously tagged protein ( Figure 1B ), which was previously reported (Lazetic et al., 2022). Thus, the intestinal-specific and epidermal-specific promoters drive expression in the expected tissues, and these results indicate that ZIP-1 ::GFP protein expression can be induced by bortezomib independently of the *zip-1* promoter. Under the control of constitutively active tissue-specific promoters, absence of ZIP-1 protein expression at baseline suggests that ZIP-1 protein is basally degraded. This degradation is mediated either directly or indirectly by the proteasome, as proteasomal blockade results in stabilization and nuclear expression of ZIP-1 .

&nbsp;

We next examined ZIP-1 ::GFP expression with these transgenes in a *zip-1 (jy13)* null mutant background. Similar to the wild-type background, *vha-6p* :: ZIP-1 ::GFP was induced in intestinal nuclei upon 4 h bortezomib treatment in a *zip-1* mutant background ( Figure 1C ). In contrast, *dpy-7p* :: ZIP-1 ::GFP was not induced upon 4 h bortezomib treatment in a *zip-1 (jy13)* mutant background ( Figure 1C ), unlike in the wild-type background. Given that endogenous *zip-1 p* :: ZIP-1 ::GFP is induced in the intestine by bortezomib, we hypothesized that bortezomib first affects the intestine, where wild-type intestinal ZIP-1 might be required to signal to the epidermis to induce epidermal ZIP-1 ::GFP expression. To investigate this possibility, we performed a time-course analysis to assess where and when ZIP-1 ::GFP is expressed after bortezomib treatment ( Figure 1D ). Here we found that *zip-1 p* :: ZIP-1 ::GFP is induced first in intestinal nuclei, and then later in epidermal nuclei. Intestinal nuclear expression is observed in roughly 50% of animals after 2 h of treatment. Similar levels of epidermal nuclear expression are only observed after 6 h of bortezomib treatment. Furthermore, all cases of epidermal nuclear expression were observed in animals that also had intestinal ZIP-1 ::GFP nuclear localization. Epidermal nuclear expression alone was not observed. This result is consistent with the model that intestinal ZIP-1 is required to send a signal to the epidermis to induce ZIP-1 ::GFP, although the specific tissue in which ZIP-1 is required for this signaling has not yet been investigated.

&nbsp;

We next tested whether these tissue-specific ZIP-1 ::GFP expression constructs could rescue mRNA expression of the IPR gene *pals-5* in *zip-1 (jy13)* mutants. *zip-1 (jy13)* mutants are defective in inducing *pals-5* mRNA after 30 minutes of bortezomib treatment. Previous results using qRT-PCR with tissue-specific RNAi suggested that ZIP-1 was required in the intestine, but not the epidermis, for this effect (Lazetic et al., 2022). &nbsp;Here we found that intestinal-specific *vha-6p* :: ZIP-1 ::GFP expression was sufficient to rescue the *pals-5* gene induction defect of *zip-1 (jy13)* mutants ( Figure 1E ). Furthermore, intestinal-specific rescue of *zip-1* resulted in significantly increased expression of another IPR gene, *skr-5 ,* over wild-type and over *zip-1 (jy13)* mutant levels. Surprisingly, epidermal-specific *dpy-7p* :: ZIP-1 ::GFP expression appeared sufficient to rescue *pals-5* induction, although the effect was not significant ( Figure 1E ). Thus, epidermal expression of ZIP-1 may be sufficient, but not required for inducing *pals-5* mRNA expression. Of note, previous work showed a partial, non-significant reduction in *pals-5* induction with epidermal-specific *zip-1* RNAi (Lazetic et al., 2022).

&nbsp;

Finally, we examined where ZIP-1 expression is sufficient to rescue the viral susceptibility of *zip-1 (jy13)* mutants. We infected animals with the Orsay virus , and then measured viral load with qRT-PCR for the Orsay virus genomic RNA1 segment. We infected *zip-1 p:: zip-1 ::gfp* animals, *zip-1 (jy13)* mutants, and *zip-1 (jy13)* mutants rescued with *vha-6p:: zip-1 ::gfp* or *dpy-7p:: zip-1 ::gfp* . Upon normalizing all data to *zip-1 p:: zip-1 ::gfp* animals, we found that intestinal-specific *vha-6p* :: ZIP-1 ::GFP expression was sufficient to rescue *zip-1 (jy13)* mutant susceptibility to viral infection. In contrast, epidermal-specific expression of ZIP-1 ::GFP was not sufficient to affect resistance to viral infection in a *zip-1 (jy13)* mutant background ( Figure 1F ).

&nbsp;

In this work, we generated tissue-specific *zip-1* expression and rescue strains to examine the regulation of protein expression and the tissue-specific effects of ZIP-1 . We demonstrated that the regulation of ZIP-1 occurs in a promoter-independent manner, suggesting that ZIP-1 is being degraded under baseline conditions. We also observed that epidermal nuclear expression of ZIP-1 is dependent on the presence of ZIP-1 in other tissues. In assessing the tissue-specific effects of ZIP-1 , we demonstrated that both intestinal and epidermal *zip-1* appear sufficient to induce mRNA expression of IPR gene *pals-5* , whereas only intestinal *zip-1* rescued viral susceptibility in *zip-1 (jy13)* mutants. However, visible expression and subsequent upregulation of ZIP-1 ::GFP in a particular tissue is not necessary for IPR activation, as *dpy-7p::* ZIP-1 ::GFP in a *zip-1 (jy13)* background displays a similar IPR activation profile following 30 minutes of bortezomib treatment to wild-type strains, despite not being visible later at 4 h. These findings support earlier findings that ZIP-1 has a functional role even when ZIP-1 ::GFP is not visible, and highlight the distinct roles of ZIP-1 at early and late stages of the IPR (Lazetic et al., 2022). Future studies could investigate where ZIP-1 is degraded basally – is it in the nucleus or the cytoplasm? What is the relationship between nuclear accumulation of ZIP-1 protein and IPR activation across tissues? Furthermore, we demonstrated that intestinal expression of *zip-1* is sufficient for defense against the Orsay virus , an intestinal pathogen. Future studies could examine defense against other intestinal pathogens like microsporidia, as well as defense against other types of pathogens, including those that infect the epidermis.&nbsp;

## Methods

**Table:** 

**Table 1**	***C. elegans* strains and plasmids **		
**Strain**	**Genotype**	**Description**	**Source**
N2	Wild type	Wild type	Troemel Lab Collection
ERT590	*zip-1 (jy13) III *	Full deletion of *zip-1*	Lažetić et al., 2022
ERT813	*zip-1 (jy132) III *	Endogenous ZIP-1 tagged with GFP	Lažetić et al., 2022
ERT1361	* knuSi895[pNU3112(vha-6p:: zip-1 ::gfp::3xFlag::eft-3 3'UTR, unc-119 (+))] II *	Intestinal expression of ZIP-1 ::GFP in wild-type background; backcrossed to N2	This study. pNU3112 plasmid details: https://invivobiosystems.benchling.com/s/seq-56QzKYzufpEX3HfhjBDb
ERT1363	* knuSi895[pNU3112(vha-6p:: zip-1 ::gfp::3xFlag::eft-3 3'UTR, unc-119 (+))] II; zip-1 (jy13) III *	Intestinal rescue of ZIP-1 ::GFP in *zip-1 (jy13)* background	This study; crossed ERT1361 to ERT590
ERT1364	* knuSi1111[pNU3111(dpy-7p:: zip-1 ::gfp::3xFlag::eft-3 3'UTR, unc-119 (+))] II *	Epidermal expression of ZIP-1 ::GFP in wild-type background; backcrossed to N2	This study. pNU3111 plasmid details: https://invivobiosystems.benchling.com/s/seq-gtvMqwCerkaGCLWt9bVV
ERT1365	* knuSi1111[pNU3111(dpy-7p:: zip-1 ::gfp::3xFlag::eft-3 3'UTR, unc-119 (+))] II; zip-1 (jy13) III *	Epidermal rescue of ZIP-1 ::GFP in *zip-1 (jy13)* background	This study; crossed ERT1364 to ERT590

**Table:** 

**Table 2**	**Primers**	
**Gene Name**	**Primer Description**	**Sequence**
*snb-1*	*snb-1* qRT-PCR Forward	ccggataagaccatcttgacg
*snb-1*	*snb-1* qRT-PCR Reverse	gacgacttcatcaacctgagc
*pals-5*	*pals-5* qRT-PCR Forward	cattggaaagcgatattgga
*pals-5*	*pals-5* qRT-PCR Reverse	tctccaggcacctatcttgtag
*skr-3*	*skr-3* qRT-PCR Forward	ccgacagccagaaacaaatca
*skr-3*	*skr-3* qRT-PCR Reverse	tctgtgatggtcttggattgac
*skr-4*	*skr-4* qRT-PCR Forward	ccgacagccagaaacaaatca
*skr-4*	*skr-4* qRT-PCR Reverse	ggtcttggattggctgatcac
*skr-5*	*skr-5* qRT-PCR Forward	cgaagagcaagatgtcaaaattg
*skr-5*	*skr-5* qRT-PCR Reverse	agaagcttggattgattggca
*cul-6*	*cul-6* qRT-PCR Forward	ctgggcttactcacaatgcc
*cul-6*	*cul-6* qRT-PCR Reverse	gcagagttggcttgctgtaa
*F26F2.1*	*F26F2.1* qRT-PCR Forward	tggaaccaggtcagagacac
*F26F2.1*	*F26F2.1* qRT-PCR Reverse	ttgtgagaatttccgcgata
*F26F2.3*	*F26F2.3* qRT-PCR Forward	ggaaagggaatgcattatgg
*F26F2.3*	*F26F2.3* qRT-PCR Reverse	ccgcacggttatttctcat
*F26F2.4*	*F26F2.4* qRT-PCR Forward	caacaatacactgcggatgg
*F26F2.4*	*F26F2.4* qRT-PCR Reverse	tcgcactgttattcatctcca
*chil-27*	*chil-27* qRT-PCR Forward	tcaagtggaggactgcaaca
*chil-27*	*chil-27* qRT-PCR Reverse	tgagttattttcggtagattccagt
Orsay RNA1	Orsay RNA1 qRT-PCR Forward	gacgcttccaagattggtattggt
Orsay RNA1	Orsay RNA1 qRT-PCR Reverse	acctcacaactgccatctaca
*zip-1 ::gfp *	*zip-1 ::gfp* genotyping Forward	cttctggccttcctcattgat
*zip-1 ::gfp *	*zip-1 ::gfp* genotyping Reverse	gtcttgtagttcccgtcatct
*zip-1 (jy13) *	*zip-1 (jy13)* genoytping Forward	cgcgattctcgtagatcaaac
*zip-1 (jy13) *	*zip-1 (jy13)* genoytping Reverse	ggagttcaaagtcgctgattg

***C. elegans* maintenance**

*C. elegans* were maintained at 20°C on *Escherichia coli* OP50-1 plated onto Nematode Growth Media (NGM) agar plates. Strains in Table 1.

***&nbsp;***

***C. elegans* strain generation**

Plasmids containing a tissue-specific promoter driving the expression of optimized *zip-1 a* with syntrons (Table 1) were used to carry out *Mos1* -mediated single copy insertion by InVivo Biosystems. Strains were backcrossed to N2 wild-type worms in the Troemel lab three times before use. Genotyping primers in Table 2.

&nbsp;

**Bortezomib treatment**

Bortezomib (Selleck Chemicals), catalog number S1013 was dissolved in DMSO to generate a 10 mM stock solution, which was diluted in M9 buffer, and added to NGM plates to achieve a final concentration of 20 μM on the plates. Synchronized L1s were grown on OP50-1 until L4, then treated with bortezomib or DMSO control. Plates were dried for 20 minutes, then incubated at 20°C for 30 minutes to 6 hours. Animals were either imaged immediately, or washed off plates with M9, then stored in Tri-reagent for RNA extraction and qRT-PCR.

**&nbsp;**

**RNA extraction and qRT-PCR**

Total RNA was isolated as previously described (Lazetic et al., 2022), then used for cDNA synthesis via the iScript cDNA kit (Bio-Rad). At least 3 independent biological replicates per group were performed in each qRT-PCR analysis using Bio-Rad CFX Connect machine with Bio-Rad CFX manager 3.1. Sequences for qRT-PCR primers are provided in Table 2. All qRT-PCR data was normalized against housekeeping gene *snb-1* , using the Pfaffl method (Pfaffl, 2001). For IPR activation assays, groups were normalized against a DMSO treated, N2 wild-type control. For Orsay virus infection, groups were normalized against an infected, *zip-1 p:: zip-1 ::gfp* control.

&nbsp;

**Orsay virus infection**

Gravid adults were bleached to obtain synchronized L1 animals, which were grown on OP50-1 until L4 and then infected with a mixture of Orsay Virus, M9 buffer, and OP50-1 culture. After 24 hours at 20°C, animals were washed off plates, and RNA was extracted for cDNA synthesis and qRT-PCR.

&nbsp;

**Imaging**

Imaging in Figure 1B was performed on a Zeiss AxioImager M1 compound microscope using ZEN Version 3.9.3. Imaging in Figure 1C was performed on a Zeiss LSM700 confocal microscope using ZEN 2010 Version 6.0.0.309. Image processing was done in Paint.NET Version 5.1.12.

&nbsp;

**Statistical analysis**

Prism 10 Version 10.5.0 (774) was used for statistical analysis.

## Data Availability

Description: pNU3111 plasmid sequence and annotation as Genbank file. Resource Type: Dataset. DOI: https://doi.org/10.22002/n687b-1hr11 Description: pNU3112 plasmid sequence and annotation as Genbank file. Resource Type: Dataset. DOI: https://doi.org/10.22002/1k6z0-hf130 Description: Raw Data Panel D. Resource Type: Dataset. DOI: https://doi.org/10.22002/azyg7-h5021 Description: Raw data panel E. Resource Type: Dataset. DOI: https://doi.org/10.22002/y8vxr-rpy89 Description: Raw data Panel F. Resource Type: Dataset. DOI: https://doi.org/10.22002/560q9-dys15
